# Effect of Carbon Nanoparticles Morphology on the Properties of Poly(styrene-*b*-isoprene-*b*-styrene) Elastomer Composites

**DOI:** 10.3390/polym15224415

**Published:** 2023-11-15

**Authors:** Xiaobing Han, Zhenhao Zhou, Jie Gao, Yuan Zhao, Tao Chen

**Affiliations:** Hubei Key Laboratory of Radiation Chemistry and Functional Materials, School of Nuclear Technology and Chemistry & Biology, Hubei University of Science and Technology, Xianning 437100, China; hanxiaobing@hbust.edu.cn (X.H.); 18872271857@163.com (Z.Z.); zhyf308@hbust.edu.cn (Y.Z.)

**Keywords:** carbon nanoparticles, morphology, property, elastomer composites

## Abstract

Though nanomaterials based on carbon have been widely used for the preparation of high-performance polymeric nanocomposites, there are few works focused on the effect of carbon nanoparticle morphology on the performance of corresponding polymer nanocomposites. Therefore, four representative carbon nanoparticles, including fullerene, carbon nanotubes, graphene, and carbon black incorporated poly(styrene-*b*-isoprene-*b*-styrene) (SIS) elastomer nanocomposites were fabricated using the solvent casting method. In addition, the effect of carbon nanoparticle morphology on the rheological, mechanical, electrical, and thermal properties of the obtained polymeric nanocomposites was systematically investigated. The results showed that the shape of carbon nanoparticles has a different effect on the properties of the obtained elastomer nanocomposites, which lays the foundation of carbon nanoparticle screening for high-performance polymer nanocomposite construction.

## 1. Introduction

Due to the combination of the plasticity of plastics and the high elasticity of rubber, a thermoplastic elastomer has been widely used in the fields of automobile, encapsulation, medical, telecommunication, and adhesives [1,2,3]. The thermoplastic elastomer can be classified as a styrene block copolymer (SBC), a thermoplastic polyurethane (TPU), a dynamically vulcanized polymer (TPV), a polyolefin (POE), and so forth [4,5]. Among the SBC thermoplastic elastomer, SIS and corresponding composites have been applied in the industrial, such as adhesives, sealants, coatings, and photo actuators. In addition, due to its high flexibility, excellent biocompatibility, chemical resistance, and good impact, SIS has been used in medicine [6,7].

**Figure 1:**
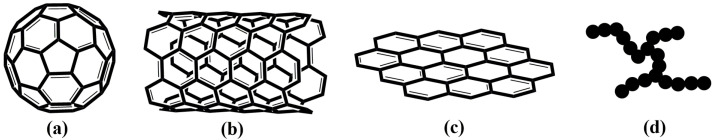
Structure of fullerene (**a**), carbon nanotube (**b**), graphene (**c**), and carbon black (**d**).

Due to its excellent electrical, mechanical, optical, and thermal properties, carbon nanoparticles such as fullerene, carbon nanotubes, graphene, and carbon black (Figure 1) have been extensively applied in fabricating high-performance polymer nanocomposites [8,9,10,11,12]. To further improve the comprehensive properties of SIS, many nanocomposite-based SIS and carbon nanoparticles were reported [13,14].

In our previous work, graphene/SIS nanocomposites were fabricated with solution casting followed by a self-assembly process [15]. In addition, the effect of π–π stacking between SIS and graphene on the different properties was investigated in detail. Graphene/SIS nanocomposites have also been prepared with solution casting for the use of packaging materials [16]; with the incorporation of layered graphene, the activation energy of oxidative degradation increased obviously. Another important application of carbon nanomaterials/SIS composites is optical actuators, including graphene/SIS and CNT/SIS nanocomposites. The graphene/SIS nanocomposites responded to IR radiation under strained conditions [17], and the maximum stress and strain were 28.34 kPa and 3.1%. Polystyrene-grafted multi-walled carbon nanotube-incorporated SIS nanocomposites were also synthesized with solution casting [18], and the optical actuation property of the obtained nanocomposites was demonstrated using atomic force microscopy. Under a power intensity of 9.6 mW with LED light, the actuation rates reached 716 nm/s and 60 nm/s under illumination and dark, respectively. However, there are no reports about the comparative study of different carbon nanoparticles for improved SIS composites.

Though single carbon nanoparticles/polymer nanocomposites have been widely studied and used, there are very few reports about the systematical investigation of the carbon nanoparticles morphology on the properties of corresponding polymer nanocomposites. Comparative investigation was conducted for CNT, graphene, and fullerene-enhanced epoxy coatings [19], and enhanced friction property was observed for all kinds of nanocomposites. However, the improvement of anti-corrosion, adhesion, and tensile was varied with the types of carbon nanoparticles. In another report, graphene, MWCNT, nanostructured CB, and amorphous CB were used for synthesizing a biodegradable and bio-based poly(butylene succinate-co-adipate) (PBSA) composite for electromagnetic application [20]. All types of nanocomposites can yield volumetric conductivity exceeding 10^−10^ S/m, but the dimensional stability, tensile, thermal conductivity, dielectric property, and transmittance vary depending on the types of carbon nanoparticles.

**Figure 2:**
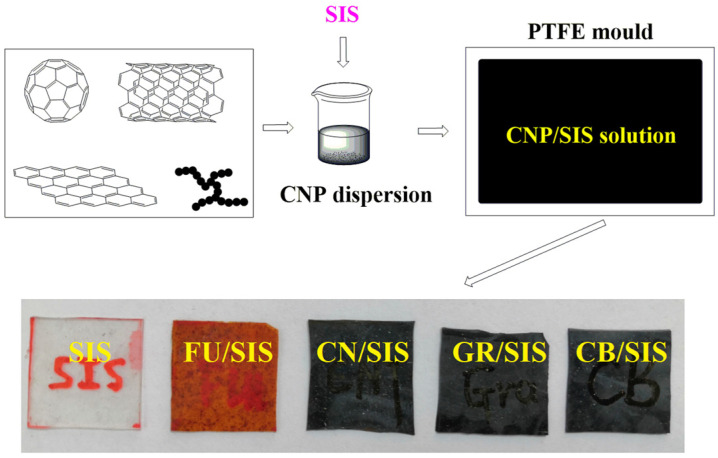
Schematic diagram of the carbon nanoparticles/SIS composites fabrication process.

As mentioned above, the morphology of the carbon nanoparticles has a great influence on the properties of the polymeric nanocomposites. To systematically assess the influence of carbon nanoparticle morphology on the performance of SIS composites, nanocomposites based on fullerene (FU), multi-walled carbon nanotubes (CN), graphene (GR), and carbon black (CB)-incorporated SIS were prepared through solution casting (Figure 2). The obtained nanocomposites were characterized by a melt flow indexer, tensile tester, ultra-high resistance tester, and thermogravimetric analysis; the different effect of carbon nanoparticle morphology on the physical properties of the synthesized composites was systematically investigated.

## 2. Materials and Methods

### 2.1. Materials

Fullerene-C_60_ (99.9%) was purchased from HWRK Chemical Co., Ltd. (Beijing, China), multi-walled carbon nanotubes (MWCNT, >95%, ID = 5–10 nm, OD = 10–20 nm, Length = 0.5–2 μm) was supplied by the Shanghai Macklin Biochemical Co., Ltd. (Shanghai, China), graphene (99.5%, D = 0.6–3 μm, thickness = 1–5 nm) and carbon black (CB, 99%, D = 30–45 nm) was obtained from Suzhou TanFeng Tech Co., Ltd. (Suzhou, China). SIS (Melt flow index = 27.50 g/10 min, Mw = 1.66 × 10^5^, PDI = 1.3, S/I = 16/84, diblock = 25%) was purchased from Xingzhi New Materials Co., Ltd. (Guangzhou, China). Chloroform was purchased from Sinopharm Chemical Reagent Co., Ltd. (Shanghai, China).

### 2.2. Fabrication of Carbon Nanoparticles/SIS Nanocomposites

Carbon nanoparticles/SIS nanocomposites were prepared with the solution casting method [15]. Typically, a 40 mg carbon nanoparticle (fullerene, MWCNT, graphene, CB) was dispersed into 12 mL chloroform assisted with an ultrasonic an hour. A 4 g SIS elastomer was put into the mixture under stirring, then the dispersion sonicating for 0.5 h and stirring for 0.5 h. The prepared dispersion was transferred into a Teflon mold; nanocomposites were achieved with evaporation and self-assembly process. Finally, the nanocomposite films were obtained and dried at 60 °C to a constant weight. The obtained nanocomposites are named FU/SIS, CN/SIS, GR/SIS, and CB/SIS.

### 2.3. Characterization

Morphology characterization of carbon nanoparticles was conducted by dropping the mixture dispersion of carbon nanoparticles onto carbon grids and determined using transmission electron microscopy (TEM, Philips TECNAI, Amsterdam, The Netherlands). The distribution state characterization of different nanoparticles in SIS was conducted using scanning electron microscopy under 10 kV (SEM, ZEISS Gemini 300, Jena, Germany). A melt indexer (MC-400A, Xiamen Min Testing Instrument and Equipment Co., Ltd., Xiamen, China) was used to reveal the melt flow index of carbon nanoparticles/SIS nanocomposites at the condition of 200 °C/5 Kg. Hardness measurements of pure SIS and SIS nanocomposites were conducted with an A Shore hardness tester (LX-A, Shenzhen Haoxinda Instrument Co., Ltd., Shenzhen, China). The tensile properties of different SIS nanocomposites were measured using a testing machine (Shimadzu AG-IC, Tokyo, Japan); five pieces of each sample were tested to obtain average values. The surface resistivity of the prepared SIS composites was measured with an ultra-high resistance tester (ST2643, Suzhou Jingge Electronic Co., Ltd., Suzhou, China). Thermogravimeter (TG) analysis was performed using a thermogravimetric analyzer (TG-209-F3, PerkinElmer, MA, USA) under the nitrogen atmosphere at a heating rate of 10 °C/min from 30 to 650 °C.

## 3. Results

### 3.1. Morphology of Carbon Nanoparticles and Nanocomposites

**Figure 3:**
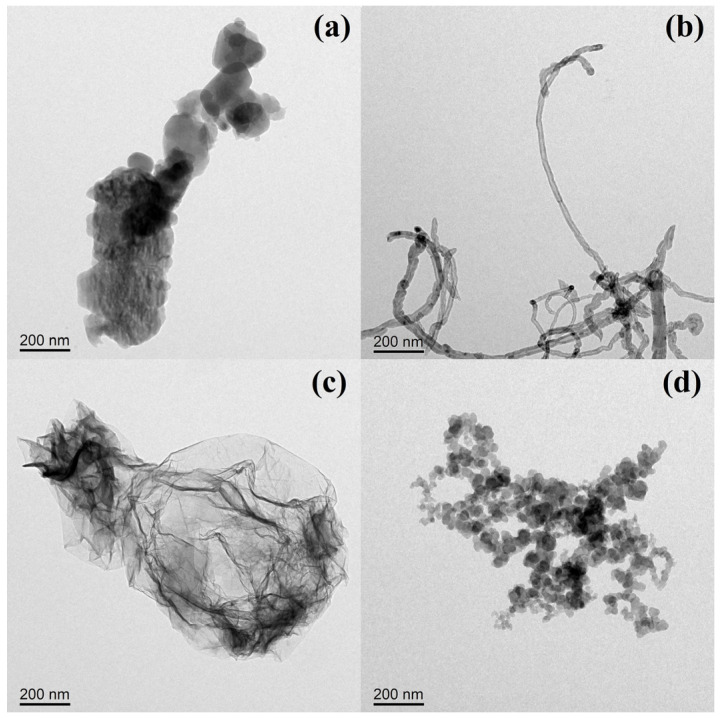
TEM images of (**a**) FU, (**b**) CN, (**c**) GR, and (**d**) CB.

To reveal the influence of carbon nanoparticle morphology on the performance of SIS nanocomposites, the morphology of FU, CN, GR, and CB was determined using transmission electron microscopy (TEM), shown in Figure 3a–d. As shown in the TEM images (Figure 3a), the FU exhibits a granular shape with an average diameter of about 200 nm. This can be ascribed to the high aggregation, leading to the random structure, which is consistent with the reported literature [21,22]. A tubulous shape was observed for the CN (Figure 3b), with a diameter of around 20 nm and a length of more than 1 μm [23]. The GR is presented in single-layered sheets (Figure 3c), with a diameter of about 800 nm and very small thickness [24]. As shown in Figure 3d, the CB shows the string of beads aggregates, which are composed of small primary particles with a diameter of around 40 nm [25]. All of the chosen carbon nanoparticles have a unique morphology; the result meets the assumption that motivated this investigation [19].

**Figure 4:**
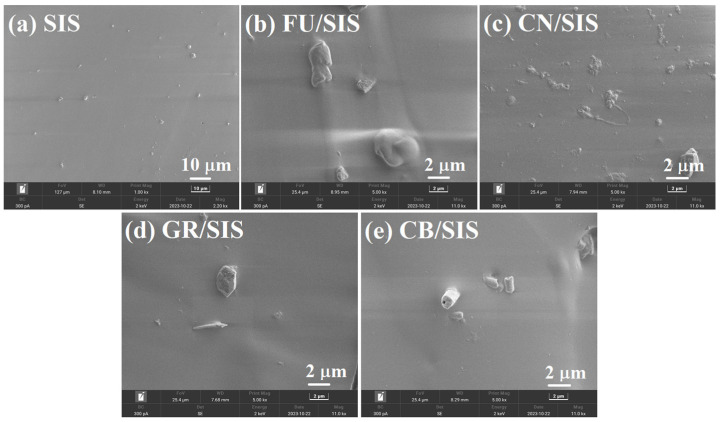
SEM images of (**a**) pure SIS and composites with (**b**) FU, (**c**) CN, (**d**) GR, and (**e**) CB.

To reveal the distribution state of different carbon nanoparticles, the cross-section morphology of pure SIS and the obtained nanocomposites was determined using scanning electron microscopy (SEM), which is shown in Figure 4a–e. As shown in Figure 4a, the pure SIS exhibits a relatively smooth surface [6]. Granular-shaped FU, tubulous-shaped CN, layered GR, and cylindrical CB appeared in the corresponding FU/SIS nanocomposites [14,17,19]. In addition, all of the carbon nanoparticles showed even distribution in the nanocomposites.

### 3.2. Melt Flow Index

**Figure 5:**
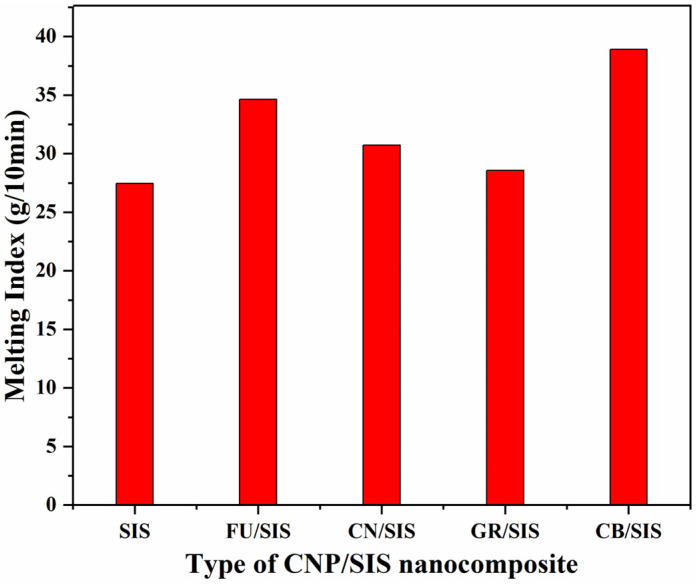
Melting index of pure SIS and different nanocomposites.

The rheological property is very important to polymeric materials, especially for the processing of elastomers; thus, the melt flow index (MFI) of the obtained nanocomposite was investigated (Figure 5). Compared with pure SIS (27.50 g/10 min), with the incorporation of different carbon nanoparticles, the MFI of all nanocomposites was increased. The value of MFI for the nanocomposites is in the order of CB > FU > CN > GR. This indicates that the mobility of the SIS chain is lightly increased, which can be ascribed to the plasticizing effect of carbon nanoparticles at low content [26,27]. The CB/SIS nanocomposite shows the highest MFI (38.88 g/10 min), revealing that the string of beads structure is favorable for the moving of the SIS chain. The GR/SIS nanocomposite shows the smallest increase in MIF (28.56 g/10 min), which can not only ascribed to the highest diameter of graphene among the four carbon nanoparticles but also closely to the strong π–π stacking interaction between graphene and SIS chains [15], which will hinder the moving of polymer chains.

### 3.3. Mechanical Property

#### 3.3.1. Shore a Hardness

**Figure 6:**
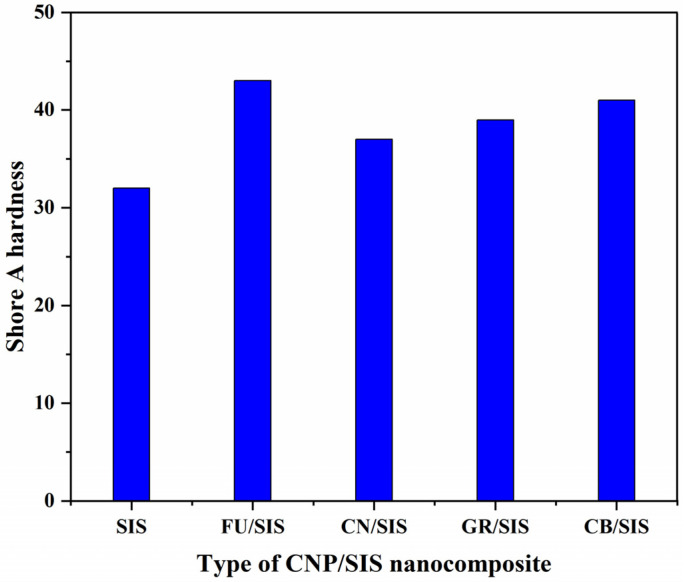
Hardness of SIS and different nanocomposites.

Figure 6 shows the effect of carbon nanoparticle morphology on the Shore A hardness of the nanocomposites. Compared with pure SIS (32), the introduction of different nanoparticles can significantly enhance the hardness of the composites [28,29,30,31], and the value of hardness for the nanocomposites is in the order of FU (43) > CB (41) > GR (39) > CN (37). The FU/SIS nanocomposites exhibit the highest hardness value, which can be ascribed to the solid structure and relatively high diameter [28]. As CB has a similar solid structure to FU, while the diameter is much lower than that of FU; therefore, the FU/SIS nanocomposites exhibit a lower hardness value [29]. For the nanofillers filled with polytetrafluoroethylene, fullerene was demonstrated to have a better hardness enhancement than graphene [30]. As the GR possesses a flexible structure with the highest diameter [30], the CN possesses a hollow and lowest diameter [31]; thus, the hardness value of GR/SIS is higher than that of CN/SIS. According to the obtained result, in the aspect of hardness enhancement, carbon nanoparticles with a solid structure and high diameter exhibit higher improvement efficiency than sheets and tubes.

#### 3.3.2. Tensile Testing

**Figure 7:**
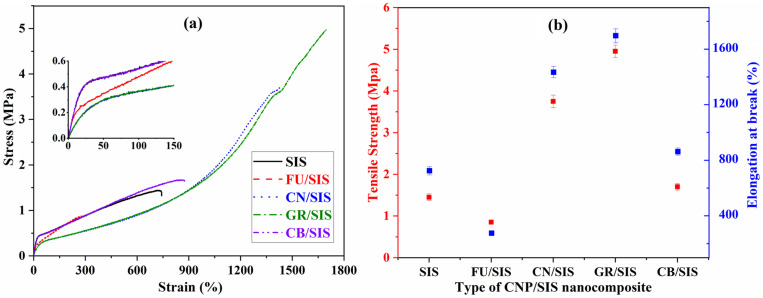
Stress–strain curves (**a**) and mechanical properties (**b**) of SIS nanocomposites.

Tensile testing was applied to assess the tensile and elongation at the break of a pure elastomer and the nanocomposites, and the stress–strain curves were presented in Figure 7a. As shown in Figure 7a, with the addition of different carbon nanoparticles, the mechanical properties of the obtained nanocomposites change variously. With the incorporation of CN, GR, and CB, the tensile strength and elongation improved, especially for the GR. However, for the FU-filled SIS, both the tensile and elongation were decreased. In addition, from the inset picture of Figure 7a, it can observed that the incorporation of carbon nanoparticles reduces the modulus of SIS at low strain. Only the CB/SIS exhibits a similar modulus of SIS; an obvious decrease of modulus was observed for other nanocomposites, especially for GR/SIS and CN/SIS composites, which can be ascribed to the plasticizing effect of GR and CN.

As shown in Figure 7b, compared with pure SIS (1.45 MPa), the tensile strength of the CN, GR, and CB-filled SIS increased to 3.75, 4.95, and 1.7 MPa, respectively, which is consistent with the reported investigation [28,29,30]. The highest enhancement efficiency was observed for the GR/SIS nanocomposites due to the highest surface area and aromatic structure of graphene, which is beneficial for the formation of strong π–π stacking interaction between graphene and SIS chains [15]. The strong π–π stacking can dissipate stress through the formed sacrificial bond, making a dramatic increase in tensile strength. However, for the FU/SIS composites, the tensile strength decreases to 0.85 MPa. A similar phenomenon was observed for fullerene-incorporated polycarbonate [32] and epoxy resin [33], which can be ascribed to the insufficient bridging effect and weak interaction between fullerene and polymeric matrix. The varies of elongation has the same phenomenon as the tensile strength, which demonstrated that for the tensile and elongation improvement of the obtained nanocomposites, layered GR and tubular CN are better than that of granular FU and CB.

### 3.4. Surface Resistivity

**Figure 8:**
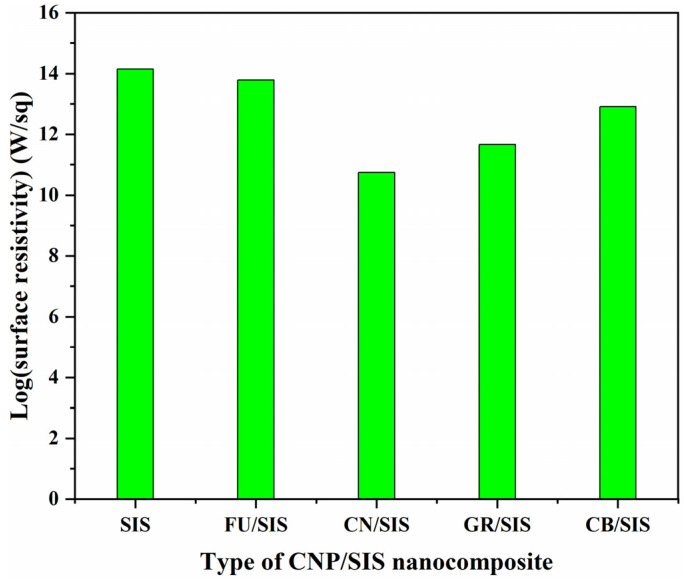
Surface resistivity of pure SIS and different nanocomposites.

Figure 8 shows the effect of carbon nanoparticles on the surface resistivity (SR) of the nanocomposites. Compared with pure SIS, the incorporation of carbon nanoparticles reduced the surface resistivity of the composites, and the value of surface resistivity for the nanocomposites is in the order of FU > CB > GR > CN. As reported in the literature, the conductivity improvement of a polymer with carbon nanoparticles can be realized using two mechanisms. On the one hand, a conductive network can be formed with high loading and even the distribution of carbon nanoparticles [34]. On the other hand, conductivity can also be improved through the hopping mechanism with isolated carbon nanoparticle incorporation [35]. As the logSR value of all the nanocomposites is more than 10, which is well above 6, the conductivity improvement of these nanocomposites can be assigned to the hopping mechanism.

Compared with pure SIS, a small decrease in the logSR value was observed for the FU/SIS composite, indicating it is hard for the electron hopping of isolated FU in the SIS matrix. As reported in the literature, graphene and carbon nanotubes show higher conductivity improvement efficiency than carbon black for polyethylene and polypropylene, respectively [34,36]. This supports the results observed in this work: the GR/SIS and CN/SIS composites have a lower surface resistivity than that of the CB/SIS composite. In theory, graphene should show higher conductivity-improvement efficiency than that of carbon nanotubes because graphene possesses higher conductivity and surface area. The GR/SIS composite has a lower conductivity than the CN/SIS composite. This can be ascribed to the fact that the CN/SIS composite can easily form a three-dimensional hopping system, but the GR/SIS can only form a two-dimensional hopping system; thus, the improvement efficiency of graphene is far lower than theoretically expected [37]. According to the obtained result, in the aspect of conductivity enhancement, carbon nanoparticles with layered or tubular structures are better than that of granular ones.

### 3.5. Thermal Stability

**Figure 9:**
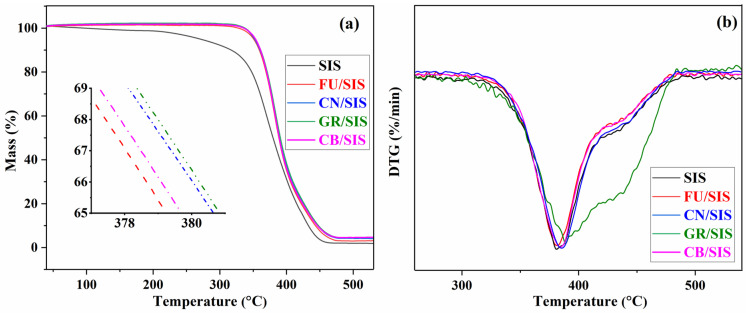
TGA (**a**) and DTGA (**b**) curves of SIS and different nanocomposites.

Thermogravimetric analysis was conducted to assess the thermal stability of SIS and the nanocomposites, and the TGA and DTGA curves of composites are present in Figure 9. As shown in Figure 9a, pure SIS exhibits the worst thermal stability; obvious thermal degradation was observed from 250 °C, and the fast degradation occurred at 350 °C [15,17]. The slight weight loss of 2.72% before 200 °C is due to the loss of physisorbed water, the weight loss of 20.13% between 200 and 350 °C can be assigned to the degradation of the isoprene segment of diblock, and weight loss after 350 °C can be attributed to the degradation of the main chain [17,38]. With the incorporation of different carbon nanoparticles, improved thermal stability was observed. From the inset picture of Figure 9a, it can be observed that the thermal stability for the composites is in the order of GR > CN > CB > FU, which is consistent with the reported investigation [39,40,41,42]. This can be assigned to the different interfacial interactions and surface areas of the nanoparticles, which can form an efficient tortuous path effect with SIS to prevent the degradation of the nanocomposite. As graphene possesses the highest surface area and aromatic structure, strong π–π stacking interaction can be formed between SIS chains and graphene, leading to the highest thermal stability of GR/SIS composites. As mentioned in the tensile strength discussion, an insufficient bridging effect and weak interaction can be formed between granular fullerene and polymeric matrix [32,33]; thus, the lowest improvement efficiency was observed for fullerene. According to the obtained result, in the aspect of thermal stability improvement, carbon nanoparticles with layered or tubular structures are also better than that of granular ones.

As shown in Figure 9b, the fastest degradation was observed around 380 °C. According to the results revealed in Figure 9a,b, the temperature of 5% weight loss (T_5%_), the temperature of the maximum rate of weight loss (T_max_), and the residue at 500 °C (R_500_) are listed in Table 1. With the incorporation of carbon nanoparticles, all of the T_5%_, T_max_, and R_500_ were improved. Importantly, 100 °C of T_5%_ was increased for all of the obtained nanocomposites due to the formation of a tortuous path effect by the incorporation of carbon nanoparticles [32,33].

## 4. Conclusions

In summary, to systematically evaluate the effect of carbon nanoparticle morphology on the physical properties of SIS nanocomposites, four kinds of SIS composites were incorporated: FU (granular), CN (tubulose), GR (layered), and CB (string of beads) were prepared through solution casting. The obtained results showed that the morphology of carbon nanoparticles has a different effect on the macroscopic properties of the prepared elastomer nanocomposites. The incorporation of carbon black can improve the mobility of the SIS, and the incorporation of fullerene can enhance the hardness of the obtained nanocomposites dramatically. In addition, adding graphene can significantly improve the tensile strength and thermal stability, while the carbon nanotubes can notably enhance the conductivity. This finding will provide a basis for the carbon nanoparticle screening in the construction of high-performance polymer nanocomposite.

## Figures and Tables

**Table 1:** Degradation parameters of SIS and carbon nanoparticles/SIS composites.

Sample	T_5%_ (°C)	T_max_ (°C)	R_500_ (%)
SIS	251.42	381.25	0.55
FU/SIS	350.19	381.89	2.82
CN/SIS	351.44	382.98	3.53
GR/SIS	351.82	383.57	3.80
CB/SIS	352.35	384.27	3.87

## Data Availability

Data produced in this study can be made available upon a reasonable request.
